# Willingness to Pay for a Hypothetical COVID-19 Vaccine in the United States: A Contingent Valuation Approach

**DOI:** 10.3390/vaccines9040318

**Published:** 2021-04-01

**Authors:** Serkan Catma, Serkan Varol

**Affiliations:** 1 Department of Business Administration, University of South Carolina Beaufort, 1 University Blvd, Hargray 224, Bluffton, SC 29909, USA; 2 Department of Engineering, Management and Technology, University of Tennessee at Chattanooga, EMCS 235, 615 McCallie Ave., Chattanooga, TN 37403, USA; Serkan-Varol@utc.edu

**Keywords:** willingness to pay, contingent valuation, COVID-19, vaccine preference

## Abstract

Our objective was to estimate the individual willingness to pay (WTP) for a COVID-19 vaccine and evaluate its predictors in the United States. A double-bounded dichotomous choice contingent valuation with open-ended question technique was implemented based on the responses to a national survey administered during the first week of November 2020. The final sample size was 1285. The results showed that individual WTP values increased with income, whether a household member had any pre-existing condition, and perceived threat of the virus. The vaccine efficacy rate and duration of protection were found to be important factors for the respondents. The mean WTP for a vaccine with a 95 percent efficacy rate and 3-year protection (US$318.76) was approximately 35 percent greater than the vaccine with a 50 percent efficacy rate and 1-year protection (US$236.85). The initial aggregate direct benefit of the current vaccination program was estimated to be between 20 and 35.6 billion US dollars depending on the vaccine protection duration.

## 1. Introduction

Coronavirus disease 2019 (COVID-19) is a natural phenomenon that has generated dire consequences and caused immediate concerns and threats to public health. It is a strain of the coronavirus family that causes respiratory and fatal infections in humans [1]. An effective vaccine is essential to mitigate the impact and spread of this dangerous virus that has resulted in over 121 million confirmed cases and nearly 2.7 million deaths globally [2]. Pharmaceutical companies work in conjunction with governments and regulatory agencies to develop vaccines that induce protective immunity. As of 1 February 2021, the United States (U.S.) Food and Drug Administration (FDA) has authorized the emergency use of both Pfizer-BioNTech and Moderna vaccines that contain mRNA, a genetic molecule that helps the body produce proteins to create antibodies against COVID-19. Both vaccines are provided at no cost to the general population under the Coronavirus Aid, Relief, and Economic Security (CARES) Act [3].

The US Government, in collaboration with pharmaceutical companies, initiated the program Operation Warp Speed (OWS) to research, develop, and effectively distribute a viable vaccine or vaccines so that herd immunity can be achieved to end this deadly pandemic. While vaccine-associated risks may be proportionately small, socio-demographic differences, the health status of individuals, and factors related to perceived risk, knowledge, and even political perceptions can influence the willingness to accept a vaccine for protection. For instance, during the 2009 H1N1 outbreak (Swine Flu), only 50 percent to 64 percent of adults in the U.S. complied with the Centers for Disease Control and Prevention’s (CDC) vaccination recommendations, mainly due to concerns about vaccine efficacy and safety [4]. It was further suggested that low uptake of vaccines was likely to be repeated due to the unpredictability of the behavior of the society. 

Understanding the factors that impact the acceptance of vaccination is a necessary and crucial step for strategic public health intervention and evaluation. However, exploring the perceived values of vaccines becomes equally important, as economic evaluation of any vaccination program requires the calculation of its social value through aggregating individual willingness to pay (WTP) values [5]. The willingness of the public to pay for a vaccine is elicited by the contingent valuation model (CVM), which has been often applied to determine individual choices and estimate the monetary values of hypothetical vaccines. WTP for a vaccine can be defined as the maximum amount of money an individual is willing to pay subject to income restrictions. Its underlying theory depends on the balance of risk and wealth and measures of how much public value the benefits they would receive from vaccination. 

The main objectives of this paper include: (1) Estimating mean and median WTP for a COVID-19 vaccine (2) analyzing the effects of socio-economic characteristics, health-related variables, perceived risk, and knowledge of the virus, and perceived effectiveness of policy measures on individual WTP values (3) assessing the differences in WTP values given the variation in vaccine efficacy rate and duration. Various efficacy rates and durations of vaccine protection were considered to assess society’s attitudes towards different risk factors.

## 2. Materials and Methods

A global survey management company, QuestionPro, was hired to administer an online questionnaire to collect information about respondents’ characteristics and their stated preferences for a hypothetical COVID-19 vaccine. The respondents were selected via convenience sampling, and an email invitation was sent to those who were 18 years of age and older. The survey took, on average, 10 min to complete and consisted of questions that intended to capture personal background information, knowledge of COVID-19, perceptions, and attitudes towards COVID-19, personal experience of the virus, and contingent valuation of a hypothetical vaccine. 

Prior to the launch of the survey, a pilot test was administrated to 100 respondents to check for the accuracy of the questionnaire and confirm the feasibility of and adjust the initial bid values. The survey was conducted during the first week of November 2020 when the news about vaccine developments were circulating in the media, but there was yet to be an FDA approved vaccine. A total of 1501 responses were collected from the residents of the US in a seven-day period.

The primary purpose of the CVM is to estimate a value of a product or service that is hypothetical in nature and/or cannot be traded in a market setting. This preferred method also allows the respondents to state their perceptions and knowledge about the good or service in question. Even though the first applications of the CVM method intended to value certain leisure activities [6,7], the broadening scope of this method has been often observed in health-related research. Moreover, the CVM has been a reliable method to assess the values of vaccines when the public is aware of their importance and effectiveness. 

Before a set of CVM questions were asked, a one-page summary outlining the meaning and relevance of different efficacy rates and duration was provided to each respondent. If a respondent accepted to pay for a vaccine out of pocket, a series of WTP questions for vaccines with 50% and 95% efficacy rates followed. It should be noted that, at the time of the survey administration, even though there were speculations about the efficacy rates of the candidate vaccines, data of their third-phase trials were still being reviewed by the FDA. The duration of vaccine protection, 1 year and 3 years, and the initial bid amounts (US$25, US$60, US$85, US$152, US$200) were randomly distributed to test the sensitivity of the elicited WTP. While this randomization provided a wider range of stated bids, the occurrence of anchoring bias was also minimized. These initial bids were structured based on the statistical analysis of the answers to the WTP questions that were collected during pre-survey testing.

While CVM can use a wide range of methods (payment cards, open-ended questions, bidding games) to help respondents state their pricing preferences, a double-bounded dichotomous choice (DBDC) with an open-ended maximum WTP question was adopted in this study, as it provides the most accurate estimates of WTP and reduces starting point bias [8,9]. As a part of this approach, if a respondent says yes to the initial bid, the next question doubles the price of the vaccine. If the respondent says no to the initial bid, the price is lowered by half in the follow-up question. Therefore, four possible WTP intervals can be obtained (“YesYes”, “YesNo”, “NoYes”, “NoNo”). This process can be repeated one more time if the answer to the follow-up question does not change. 

Table 1 shows the distribution of Yes and No answers to the initial and follow-up questions for Vaccine-1 and Vaccine-2. For instance, if the respondent rejected the first bid but accepted the second bid, the response was marked as “NoYes”. 41.7 percent of the respondents stated that they would not be willing to pay for any of the vaccines. As expected, the ratio of respondents who agreed to purchase a vaccine and the ratio of “YesYes” to total responses were higher for the vaccine with the highest efficacy rate (vaccine-2). 

Regardless of the answers to the last WTP question, each respondent was asked to state their maximum willingness to pay for the vaccine in question. However, the interval-censored observations were used in the statistical model as using open-ended questions cause significant biases [10,11] and inflated WTP values [12]. In other words, if a respondent accepted to pay US$50 for a vaccine but refused to pay US$100, the WTP could be assumed to be in the range of US$50 and US$100. If the same respondent’s answer to the open-ended question was greater than US$100, this contradictory sample was excluded from further consideration due to the conflicting nature of the answers. Protest answers where respondents said no to all the WTP questions and stated 0 maximum willingness to pay were also censored. After the elimination of inconsistent responses, protest answers, and observations with missing values, the outliers were identified using box-plot and q-q plot methods thus removed from the final dataset. 

Parametric estimation of the interval regression model was the preferred method in this study as it results in a more accurate estimation of mean WTP and is more suitable for the incorporation of socio-demographic factors [13]. Numerous classical distributions have been presented for the parametric estimation of DBDC data. While both logistic and normal distributions have been extensively used in the literature [14,15], the log-logistic distribution is an appropriate choice when choice responses are interval-censored, and WTP values are non-negative [16]. 

## 3. Results

### 3.1. Respondent Characteristics

In total, 1285 respondents completed questionnaires containing measures of COVID-19 related attitudes and beliefs including socio-demographics, health-related, knowledge, perceived risk, and effectiveness of policy measure variables. The measurement of knowledge consisted of four separate questions, but for simplicity, the correct answers were aggregated by assigning equal weight to each correct response. Table 2 defines and provides summary statistics for the variables considered in this study.

The dichotomous socio-demographic variables represented variation in age, gender, race, education, income, and marital status data. The health-related components were observed through the reflection of the health status of the respondents including pre-existing and post-infection status. Additionally, the knowledge factors pertaining to contraction and symptoms of COVID-19, virus prevention measures, and contagiousness rates in the U.S were assessed to gauge the community’s knowledge of COVID-19. A measure of risk assessment and perception was administrated and constructed based on the perceived seriousness and threat of the disease and the pandemic. The effectiveness of policy measures was recorded in reference to the responses of whether event and venue closings, mandatory stay at home orders, and the mandatory wearing of face masks were effective.

### 3.2. Factors Affecting WTP for COVID-19 Vaccine

We constructed and assessed three empirical models in this study. The main model (Model-1) included socio-demographic and other relevant characteristics to identify the factors that significantly contribute to the value of the COVID-19 vaccine with a 95 percent efficacy rate (Table 3). The inclusion of these variables aimed to lower the negative effects of the omitted-variable bias. The estimated mean and median WTP values are based on Model-2 and Model-3 results (Appendix A, Table A1) and discussed later in the paper. 

Among the respondents who expressed willingness to pay for a vaccine with a 95 percent efficacy rate, gender, age, and income were the only socio-demographic characteristics that had significant impact on WTP. Among the respondents, an annual income of less than US$60,000 was associated with lower WTP than an income of US$100,000. The only age interval that was associated with lower WTP was 46–55. Males’ higher WTP for a COVID-19 vaccine was consistent with the finding of a similar study that investigated the value of a hypothetical COVID-19 vaccine in Ecuador [17].

Health-related characteristics included the health status of the respondents and household members and their experiences with COVID-19. The regression results indicated that having health insurance and living with someone with a pre-existing condition were positively related to WTP. The scoring system to assess the knowledge of COVID-19 was found to have no statistical impact on WTP, which was found to be the case in Ecuador and Chile [17,18].

“Threat” and “worried” were used as the indicators of perceived risk for COVID-19. The respondents who agreed that the pandemic was a serious public threat had significantly higher WTP for the hypothetical vaccine. Additionally, certain policy measures to combat the COVID-19 pandemic were included in the model since these measures have important political dimensions, especially in the US. The respondents who found closing all shops except supermarkets and pharmacies effective were associated with higher WTP. 

The empirical model had a good statistical fit (likelihood ratio test = 106.64, *p* = 0.000). To further confirm the wellness of the model performance, we followed a similar three-step validation process suggested by Palanca-Tan [13]. First, income was found to be statistically significant and positively related to WTP, a common and expected finding in the past CVM studies [17,18,19,20]. Second, the log of the bid had a negative sign with a *p*-value of 0.000. In other words, a respondent’s probability of rejecting a bid was found to be rising as the bid amount was increased. Third, a scope test was performed by examining the relationship between the duration of the efficacy of the vaccine and WTP. The vaccine with longer protection from the disease was associated with higher WTP than the vaccine with shorter protection. This finding is especially important, as the efficacy durations of current vaccines are yet to be known.

### 3.3. Mean and Median WTP

In model 2 and model 3, only the socio-demographic characteristics were included. The primary purpose of constructing these models was not to identify the factors that would impact WTP, but to estimate the mean and median WTP values for vaccine-1 and vaccine-2. Table 4 presents the parametric estimates of the mean and median WTP values for vaccines with different efficacy rates and duration. It should be noted that the confidence interval for the estimates of mean and median values is 95 percent and based on the bootstrap method with 1000 replications.

It can be readily observed that the efficacy rate played an important role in determining the value of the vaccine. Respondents on average would be willing to pay US$293 for a vaccine that had a 95 percent efficacy rate. Median WTP for a vaccine with a 95 percent efficacy rate (US$210.32) was approximately 35 percent higher than the vaccine with almost half the efficacy rate (US$154.21). A similar finding was observed in a recent study in which respondents in Australia would be willing to pay $23.92 for a percentage increase in COVID-19 vaccine efficacy [21]. 

After a vaccine with longer protection (three years) was introduced, the median WTP increased to US$236.81 (from US$185.28). Among all the four different vaccine scenarios, while the vaccine with a 3-year efficacy duration and a 95 percent efficacy rate had the highest mean and median values (US$318.76 and US$236.81), the vaccine with a 50 percent efficacy rate and 1 year protection was estimated to have the lowest (US$236.85 and US$154.21) values.

## 4. Discussion

A CVM was adopted to assess the value of a hypothetical COVID-19 vaccine and determine the associated exogenous variables in the U.S. Even though numerous studies investigated the willingness to accept (WTA) for a COVID-19 vaccine in the United States [22,23,24], to our best knowledge, this is one of the first studies that projected the value of a hypothetical COVID-19 vaccine using the CVM. 

The survey data show that approximately 42 percent of the respondents would not buy a COVID-19 vaccine if the cost of the vaccine is fully paid by the respondents. However, this estimate should be interpreted with caution as the information about free vaccination, at least in 2021 or during the pandemic, was known by most Americans during the time of the survey administration. For instance, Reiter et al. [22] found that the WTA for a COVID-19 vaccine was nearly 70 percent in the US, but only 35 percent of the participants would be willing to pay US$50 or more for it. According to a survey administered by the Pew Research Center in December 2020 [25], 39 percent of the Americans stated that they “definitely or probably” would not get a coronavirus vaccine. Unlike the higher vaccine acceptance rates in China, Ecuador, and Chile [17,18,26], the vaccine resistance, budgetary concerns, and the expectation that the vaccine would be fully subsidized by the government most likely contributed to lower acceptance rates. 

**Figure 1:**
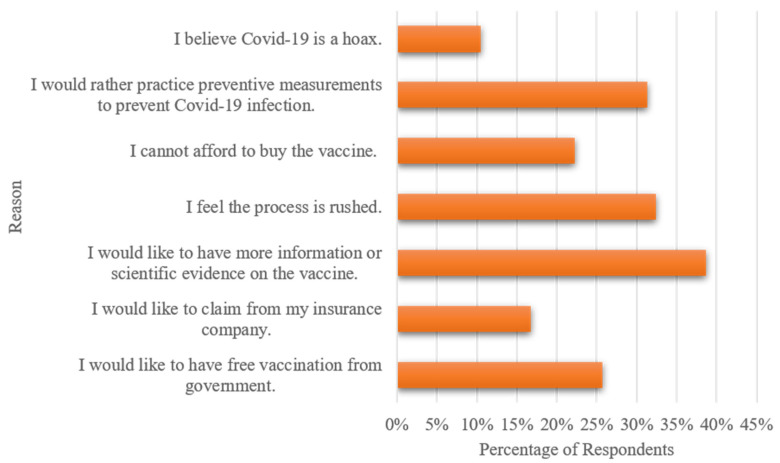
Reasons for rejecting a COVID-19 vaccine.

Figure 1 presents the reasons for rejecting a COVID-19 vaccine. The uncertainty and knowledge deficiencies signify greater perceived risk-controlled via emotional appraisals and cognitive skills. The negative association of perceived risk, awareness, knowledge gap, and willingness to participate in vaccination was echoed in our survey responses. Even as cases surge throughout the globe, the pandemic denial is a reality, where nearly one-tenth of the participants expressed that COVID-19 was a perpetrated hoax. The lack of public knowledge about the disease or unveiled scientific evidence constituted the leading reason why people were rejecting the vaccine. This was followed by the preference to practice preventive measurements, which could be possibly related to the greater perceived health risk factors or the perceived cost and lack of benefit association. The economic aspects such as concerns over the vaccine’s affordability, expectations of a free vaccination from the government, and seeking health insurance coverage stood out as substantial obstacles in the way of accepting self-paying vaccines. As confirmed by similar studies conducted in Indonesia [27], Ecuador [17], Chile [18], and Malaysia [28], pandemic vaccination programs should consider the economic status of lower income groups, as lower WTP and refusal to be vaccinated are partly due to financial reasons. 

One interesting finding of this study is that while the health status of the respondents did not appear to influence the WTP, living with someone with a pre-existing condition was found to be more important in explaining the variation in WTP. This may echo the altruistic nature of the vaccination and decision to be vaccinated, especially when the family members are involved. Considering the lower acceptance rates of vaccines in the United States, unless cooperative altruism is widely adopted and acknowledged, the roadblocks to achieving herd immunity may be harder to remove. 

As the estimated vaccine efficacy rate was widely recognized by the public during the data collection process, the vaccine with a 95 percent efficacy rate had been the center of this study’s focus. As predicted, respondents were willing to pay significantly less for a vaccine that had almost half of the efficacy rate. The duration of protection was entered into the interval regression model and was found to increase individuals’ perceived value of the vaccine. This is especially important because there is currently not enough data regarding how long Pfizer/Biotech and Moderna vaccines protect the individuals from COVID-19. Potential benefits of the vaccine would increase significantly as it provides longer protection, and the regression results confirmed the realization of these private benefits by the survey respondents. The optimal vaccination rate to reach herd immunity can be achieved if the higher WTP values are translated into higher WTA.

Comparison of the estimated values of WTP can help us understand the important geographical and other differences that may impact the perceived value of vaccines. Sarasty et al. [17] assessed the value of a hypothetical COVID-19 vaccine with different efficacy rates and duration in Ecuador and found the mean and median WTP values to increase with higher efficacy rate and duration. Their parametric mean estimate of a vaccine with a 98 percent efficacy rate and 1-year duration is an important reference point, as it is almost identical to one of the four scenarios (1 year and a 95 percent efficacy rate) we investigated. They found the mean WTP to be US$327.81, which is slightly higher than our estimate of US$264.97. Dong et al. [26] also found that the respondents in China were willing to pay a premium for a higher efficacy rate and longer protection from the virus. Their estimate of mean WTP for a 90 percent efficacy rate and 18 months of protection was 1948 CNY (US$301.36). Garcia and Cerda [18] conducted a similar study in Chile and though the effectiveness of the hypothetical vaccine was not disclosed in the study, their mean estimate was within the range of US$169.92 and US$184.72.

It is also worthwhile to mention similar studies that estimated significantly lower WTP values. For instance, the mean WTP for a hypothetical COVID-19 vaccine was US$57.2 and US$30.66 in Indonesia and Malaysia, respectively [27,28]. These discrepancies among countries should be evaluated within the context of macroeconomic and socio-demographic differences. In other words, it is important to evaluate the mean and median WTP projections based on economic, demographic, and social norms that are present where the surveys are administered. Additionally, the timing of the data collection is equally critical, as the severity of the pandemic has shown significant variations over time globally.

Estimated mean and median WTP values reflect the private values of disease prevention. Even though the U.S. government announced that the vaccine would be provided free of charge to the American people during the pandemic, identifying the private benefit of getting the vaccine is a crucial step in assessing the overall cost–benefit relationship of any vaccination program. As of January 2021, the U.S. government has secured the right to purchase 200 million doses of Pfizer/BionTech and Moderna vaccines under the program Operation Warp Speed (OWS), a public–private partnership. OWS’s main objective of producing and delivering vaccines to American people is estimated to cost 10 billion dollars to the federal government [29]. According to the former Secretary of the Pennsylvania Department of Health, Dr. Rachel Levine’s testimony at the Senate hearing in December 2020 [30], the States may have to bear an additional cost of 8 billion dollars. Assuming that the vaccine, at least initially, will be available to those who are 18 years of age and older, CDC’s estimate of flu vaccination rate among adults can be used as a reference point. During the 2019–2020 season, the flu vaccination rate among adults was 48.4 percent in the United States [31]. According to the US Census [32], the number of people who were 18 and older was 255,200,373 in 2019. If the flu vaccination rate is used as a reference point, the total number of people getting the COVID-19 vaccine would be 123,516,980. Table 5 reports the total private value of preventing COVID-19 through vaccination by efficacy duration using the parametric estimates of the mean and median WTP values. 

The above values are conservative estimates as lower vaccine acceptance rate and lower bound of mean and median WTP values were used in the calculations. If the COVID-19 vaccine provides protection for one year, the estimated private value of preventing the virus would be between 20 and 29 billion US dollars. This estimate increases significantly to the range of 25.7 and 35.6 billion US dollars if the vaccine protection lasts 3 years. Even though there are other direct and indirect costs and benefits of vaccinating the US population, documenting the private value can be a useful first step to assess the effectiveness of the current vaccination efforts. However, a more accurate estimation of total private value can be obtained by targeting a study sample that characterizes the US population. Therefore, one should be cautious interpreting these values. 

It is worth mentioning the limitations and strengths of this study. Even though the CVM provides an accurate assessment of WTP values, respondents can always understate or overstate their perceived value of a hypothetical vaccine when there is a hypothetical outbreak. However, the circumstances are unique in a way that during the administration of this survey, the news about vaccine development were circulating in the media, but none of the candidate vaccines completed the formal approval process. Responses to survey questions about a quasi-hypothetical vaccine during a real outbreak may provide unique and perhaps more realistic estimates of vaccine valuation. However, the discussions about the pricing of the vaccines in preparation, the governmental decision to fully subsidize the vaccine cost, and information about the potential vaccines may have influenced the respondents’ stated preferences. 

## 5. Conclusions

The COVID-19 pandemic has been causing immense suffering and wreaking economic havoc across the globe. Tremendous resources have been allocated by public and private entities to end this deadly outbreak. Our estimates of the WTP values are important indicators of the benefits individuals receive from vaccination. These results can also be used to evaluate the costs and benefits of public and private vaccination efforts. 

## Figures and Tables

**Table 1:** Percentages of yes and no answers to willingness to pay (WTP) questions.

Question	Response	Percentage
If all the vaccines must be paid fully by you, which vaccine would you buy? (*N* = 1285)		
	Vaccine-1	6.87%
	Vaccine-2	51.43%
	Neither	41.70%
If only vaccine-1 is available, would you be willing to purchase vaccine-1? (*N* = 1285)		
	Yes	39.35%
	No	60.65%
Would you be willing to pay $X per two doses of COVID-19 Vaccine-1 for yourself? (*N* = 506)		
	NoNo	8.89%
	NoYes	18.38%
	YesNo	46.64%
	YesYes	26.09%
If only vaccine-2 is available, would you be willing to purchase vaccine-2? (*N* = 1285)	Yes	47.51%
	No	52.49%
Would you be willing to pay $X per two doses of COVID-19 Vaccine-2 for yourself? (*N* = 611)		
	NoNo	2.78%
	NoYes	18.00%
	YesNo	46.15%
	YesYes	33.06%

**Table 2:** Variable definitions and descriptive statistics (*N* = 1285).

Variable	Definition	n (%)	Mean
Socio-demographics			
Age	Age of the respondent:		
	1 if between 18–24	180 (14%)	
	2 if between 25 and 35	303 (25%)	
	3 if between 36–45	207 (16%)	
	4 if between 46–55	182 (14%)	
	5 if between 56–65	187 (15%)	
	6 if more than 65	226 (18%)	
Female	1 if respondent is female, 0 = male	904 (70%)	
White	1 if respondent is White, 0 = otherwise	984 (77%)	
Education	1 if respondent is a college graduate, 0 = otherwise	688 (54%)	
Income	Annual family income:		
	1 if <$20,000	293 (23%)	
	2 if between $20,000–$39,999	279 (22%)	
	3 if between $40,000–$59,999	264 (21%)	
	4 if between $60,000–$79,999	169 (13%)	
	5 if between $80,000–$99,999	95 (7%)	
	6 if more than $100,000	185 (14%)	
Marital	1 if respondent is married, 0 = otherwise	561 (43%)	
Health-related variables			
Health Insurance	1 if respondent has health insurance, 0 = otherwise	1169 (91%)	
Health Status of Respondents	1 if respondent’s perceived health status is excellent, very good, or good, 0 = otherwise	1084 (84%)	
Pre-existing Condition-Respondent	1 if respondent has at least one pre-existing condition, 0 = otherwise	479 (37%)	
Pre-existing Condition-Household	1 if respondent is living with someone who has at least one pre-existing condition, 0 = otherwise	446 (35%)	
Positive COVID-19 Test—Respondent	1 if respondent got tested positive for COVID-19; 0 = otherwise	85 (7%)	
Positive COVID-19 Test—Household	1 if a household member got tested positive for COVID-19; 0 = otherwise	112 (9%)	
Knowledge	The sum of correct answers to the questions about the following sections (maximum of 22 points)		
Symptoms of COVID-19	Symptoms of COVID-19 (maximum of 11 points)		7.60
Ways of Contracting COVID-19	Ways through which COVID-19 virus is contracted (maximum of 3 points)		2.31
Prevention of COVID-19	Measures to prevent spread of COVID-19 (maximum of 7 points)		6.09
Number of people infected	“How many people are infected with COVID-19 virus in the United States?” 1 if respondent picked the right answer; 0 = otherwise	350 (27%)	
Perceived risk			
Threat	“How serious of a public health threat do you think the coronavirus is now?” 1 if respondent answered “strongly agree” or “somewhat agree”; 0 = otherwise	942 (73%)	
Worried	“How worried are you about getting COVID-19?” 1 if respondent answered “very worried” or “worried”; 0 = otherwise	602 (47%)	
Effectiveness of policy measures			
Close schools and daycares	1 if respondent thinks “closing schools and daycares” is an effective or highly effective policy measure, 0 = otherwise	635 (49%)	
Close all shops except for supermarkets and pharmacies	1 if respondent thinks “closing all shops except for supermarkets and pharmacies” is an effective or highly effective policy measure, 0 = otherwise	589 (46%)	
Mandatory stay at home order	1 if respondent thinks “obliging everyone who does not work in a crucial professional group to stay at home except to do basic shopping or because urgent medical care is required” a highly effective or effective policy measure, 0 = otherwise	732 (57%)	

**Table 3:** Interval regression results of WTP for a COVID-19 vaccine.

Variable	β	Standard Error
Bid (in log form)	−1.768 ***	0.076
Age		
18–24	−0.129	0.305
25–35	−0.389	0.26
36–45	−0.442	0.276
46–55	−0.763 ***	0.293
56–65	−0.257	0.261
>65	reference	
Female (female = 1, male = 0)	−0.379 **	0.17
White (white = 1, others = 0)	0.2	0.197
Education (graduated from college = 1, others = 0)	0.233	0.172
Income		
<$20,000	−0.871 ***	0.308
$20,000–$39,999	−0.877 ***	0.276
$40,000–$59,999	−0.52 **	0.26
$60,000–$79,999	−0.221	0.287
$80,000–$99,999	−0.158	0.341
>$100,000	reference	
Health Insurance (insured = 1; uninsured = 0)	0.533 *	0.324
Health Status of Respondents (excellent, very good, good = 1; others = 0)	−0.124	0.247
Pre-existing Condition-Respondent (yes = 1, no = 0)	0.131	0.185
Pre-existing Condition-Household (yes = 1, no = 0)	0.260 *	0.167
Positive COVID-19 Test—Respondent (yes = 1, no = 0)	−0.11	0.364
Positive COVID-19 Test—Household (yes = 1, no = 0)	0.26	0.309
COVID-19 knowledge (maximum score of 22 points)	0.034	0.02
Threat (somewhat agree or strongly agree = 1; others = 0)	0.839 ***	0.26
Worried (very worried or worried = 1; others = 0)	0.26	0.174
Close schools and daycares (highly effective or effective = 1, others = 0)	−0.081	0.199
Close all shops except for supermarkets and pharmacies (highly effective or effective = 1, others = 0)	0.446 **	0.201
Mandatory stay at home order (highly effective or effective = 1, others = 0)	−0.157	0.202
Mandatory wearing of face masks (highly effective or effective = 1, others = 0)	0.3	0.244
Duration (1–3 years)	0.204 ***	0.076
Intercept (constant)	7.415 ***	0.752
*N*	611	
Log likelihood	−842.59	
LR test	106.64	
*p* value	0.000	

* Significant at 90% level; ** Significant at 95% level; *** Significant at 99% level.

**Table 4:** Parametric estimates of mean and median WTP for a COVID-19 vaccine.

Vaccine	WTP
50% efficacy rate	
Mean	$236.85 ($216.09–$257.44)
Median	$154.21($138.83–$171.48)
95% efficacy rate	
Mean	$293.51 ($270.93–$314.88)
Median	$210.32($191.42–$231.39)
Duration	
1 year efficacy duration	
Mean	$264.97 ($235.56–$295.75)
Median	$185.28 ($162.53–$210.84)
3 year efficacy duration	
Mean	$318.76 ($288.46–$352.01)
Median	$236.81 ($208.17–$270.74)

**Table 5:** Private value of preventing COVID-19 through vaccination.

Vaccine Type	Private Value
Vaccine with 1-year efficacy duration	
Lower Bound based on Mean WTP	US$29,095,659,809
Lower Bound based on Median WTP	US$20,075,214,759
Vaccine with 3-year efficacy duration	
Lower Bound based on Mean WTP	US$35,629,708,051
Lower Bound based on Median WTP	US$25,712,529,727

## Data Availability

The data presented are available on request from the corresponding author.

## Appendix A

**Table A1:** Regression results of Model-2 (50% efficacy rate) and Model-3 (95% efficacy rate).

Variable	Model-2		Model-3	
	β	Std Error	β	Std Error
Bid (in log form)	−1.566 ***	0.073	−1.638 ***	0.07
Age				
18–24	−0.512 *	0.297	−0.353	0.281
25–35	−0.386	0.259	−0.591 **	0.238
36–45	−0.477 *	0.28	−0.43 *	0.26
46–55	−0.79 ***	0.304	−0.875 ***	0.28
56–65	−0.219	0.276	−0.29	0.255
>65	reference			
Female (female = 1, male = 0)	0.115	0.176	−0.058	0.16
White (white = 1, others = 0)	0.371*	0.206	0.197	0.193
Education (graduated from college = 1, others = 0)	0.162	0.176	0.253	0.17
Income				
<$20,000	−0.766 **	0.317	−0.764 ***	0.292
$20,000–$39,999	−0.587 **	0.29	−0.826 ***	0.265
$40,000–$59,999	−0.665 **	0.27	−0.575 **	0.252
$60,000–$79,999	−0.311	0.288	−0.181	0.28
$80,000–$99,999	−0.087	0.358	−0.303	0.334
>$100,000	reference		0.157 ***	0.051
Intercept (constant)	8.209 ***	0.421	9.347 ***	0.517
*N*	506		611	
Log likelihood	−768.81		−876.67	
LR test	30.48		38.48	
*p* value	0.004		0.000	

* Significant at 90% level. ** Significant at 95% level. *** Significant at 99% level.

## References

[1] Docea A.O., Tsatsakis A., Albulescu D., Cristea O., Zlatian O., Vinceti M., Moschos S.A., Tsoukalas D., Goumenou M., Drakoulis N. (2020). A new threat from an old enemy: Re-emergence of coronavirus (Review). Int. J. Mol. Med..

[2] World Health Organization Coronavirus (COVID-19) Dashboard. https://covid19.who.int/.

[3] Centers for Disease Control and Prevention Key Things to Know about COVID-19 Vaccines. https://www.cdc.gov/coronavirus/2019-ncov/vaccines/keythingstoknow.html.

[4] Ravert R.D., Fu L.Y., Zimet G.D. (2012). Reasons for Low Pandemic H1N1 2009 Vaccine Acceptance within a College Sample. Adv. Prev. Med..

[5] Asgary A. (2012). Assessing households’ willingness to pay for an immediate pandemic influenza vaccination programme. Scand. J. Public Health.

[6] Davis R.K. (1963). The Value of Outdoor Recreation: An Economic Study of the Marine Woods. Ph.D. Thesis.

[7] Cicchetti C.J., Smith V.K. (1973). Congestion, quality deterioration, and optimal use: Wilderness recreation in the Spanish peaks primitive area. Soc. Sci. Res..

[8] Boyle K.J., Champ P.A., Boyle K.J., Brown T.C. (2017). Contingent valuation in practice. A Primer on Nonmarket Valuation.

[9] Neumann P.J., Cohen J.T., Hammitt J.K., Concannon T.W., Auerbach H.R., Fang C., Kent D.M. (2012). Willingness-to-pay for predictive tests with no immediate treatment implications: A survey of US residents. Health Econ..

[10] Sun C., Yuan X., Xu M. (2014). The public perceptions and willingness to pay: From the perspective of the smog crisis in China. J. Clean. Prod..

[11] Khan N.I., Brouwer R., Yang H. (2014). Household’s willingness to pay for arsenic safe drinking water in Bangladesh. J. Environ. Manag..

[12] Heinzen R.R., Bridges J.F.P. (2008). Comparison of four contingent valuation methods to estimate the economic value of a pneumococcal vaccine in Bangladesh. Int. J. Technol. Assess. Health Care.

[13] Palanca-Tan R. (2008). The demand for a dengue vaccine: A contingent valuation survey in Metro Manila. Vaccine.

[14] Al-Shomrani A.A., Shawky A.I., Arif O.H., Aslam M. (2016). Log-logistic distribution for survival data analysis using MCMC. SpringerPlus.

[15] Bateman I., Carson R., Day B., Hanemann M., Hanley N., Hett T., Jones-Lee M., Loomes G., Mourato S., Özdemirog lu E. (2002). Economic Valuation with Stated Preference Techniques: A Manual.

[16] Aizaki H., Nakatani T., Sato K. (2014). Stated Preference Methods Using R.

[17] Sarasty O., Carpio C.E., Hudson D., Guerrero-Ochoa P.A., Borja I. (2020). The demand for a COVID-19 vaccine in Ecuador. Vaccine.

[18] García L.Y., Cerda A.A. (2020). Contingent assessment of the COVID-19 vaccine. Vaccine.

[19] Lin P.-J., Saret C.J., Neumann P.J., Sandberg E.A., Cohen J.T. (2016). Assessing the Value of Treatment to Address Various Symptoms Associated with Multiple Sclerosis: Results from a Contingent Valuation Study. PharmacoEconomics.

[20] Lin C.-T., Huang Y.-S., Liao L.-W., Ting C.-T. (2020). Measuring Consumer Willingness to Pay to Reduce Health Risks of Contracting Dengue Fever. Int. J. Environ. Res. Public Health.

[21] Borriello A., Master D., Pellegrini A., Rose J.M. (2021). Preferences for a COVID-19 vaccine in Australia. Vaccine.

[22] Reiter P.L., Pennell M.L., Katz M.L. (2020). Acceptability of a COVID-19 vaccine among adults in the United States: How many people would get vaccinated?. Vaccine.

[23] Kreps S., Prasad S., Brownstein J.S., Hswen Y., Garibaldi B.T., Zhang B., Kriner D.L. (2020). Factors Associated with US Adults’ Likelihood of Accepting COVID-19 Vaccination. JAMA Netw. Open.

[24] Malik A.A., McFadden S.M., Elharake J., Omer S.B. (2020). Determinants of COVID-19 vaccine acceptance in the US. EClinicalMedicine.

[25] Pew Research Center (2020). Intent to Get a COVID-19 Vaccine Rises to 60% as Confidence in Research and Development Process Increases. https://www.pewresearch.org/science/2020/12/03/intent-to-get-a-covid-19-vaccine-rises-to-60-as-confidence-in-research-and-development-process-increases/.

[26] Dong D., Xu R.H., Wong E.L., Hung C., Feng D., Feng Z., Yeoh E., Wong S.Y. (2020). Public preference for COVID-19 vaccines in China: A discrete choice experiment. Health Expect..

[27] Harapan H., Wagner A.L., Yufika A., Winardi W., Anwar S., Gan A.K., Setiawan A.M., Rajamoorthy Y., Sofyan H., Vo T.Q. (2020). Willingness-to-pay for a COVID-19 vaccine and its associated determinants in Indonesia. Hum. Vaccines Immunother..

[28] Wong L.P., Alias H., Wong P.-F., Lee H.Y., Abubakar S. (2020). The use of the health belief model to assess predictors of intent to receive the COVID-19 vaccine and willingness to pay. Hum. Vaccines Immunother..

[29] United States Government Accountability Office (2020). COVID-19 Federal Efforts Accelerate Vaccine and Therapeutic Development, but More Transparency Needed on Emergency Use Authorizations. https://www.gao.gov/assets/720/710691.pdf.

[30] United States Senate Committee on Commerce, Science, and Transportation (2020). The Logistic of Transporting a COVID-19 Vaccine. https://www.commerce.senate.gov/services/files/1E076DB3-C500-451D-B0E6-1723FFF9C719.

[31] Centers for Disease Control and Prevention (2020). Flu Vaccination Coverage, United States, 2019–2020 Influenza Season. https://www.cdc.gov/flu/fluvaxview/coverage-1920estimates.htm.

[32] United States Census Bureau (2020). National Population by Characteristics: 2010–2019. https://www.census.gov/data/tables/time-series/demo/popest/2010s-national-detail.html.

